# Preoperative risk stratification in endometrial cancer (ENDORISK) by a Bayesian network model: A development and validation study

**DOI:** 10.1371/journal.pmed.1003111

**Published:** 2020-05-15

**Authors:** Casper Reijnen, Evangelia Gogou, Nicole C. M. Visser, Hilde Engerud, Jordache Ramjith, Louis J. M. van der Putten, Koen van de Vijver, Maria Santacana, Peter Bronsert, Johan Bulten, Marc Hirschfeld, Eva Colas, Antonio Gil-Moreno, Armando Reques, Gemma Mancebo, Camilla Krakstad, Jone Trovik, Ingfrid S. Haldorsen, Jutta Huvila, Martin Koskas, Vit Weinberger, Marketa Bednarikova, Jitka Hausnerova, Anneke A. M. van der Wurff, Xavier Matias-Guiu, Frederic Amant, ENITEC Consortium, Leon F. A. G. Massuger, Marc P. L. M. Snijders, Heidi V. N. Küsters-Vandevelde, Peter J. F. Lucas, Johanna M. A. Pijnenborg

**Affiliations:** 1 Department of Obstetrics and Gynaecology, Radboud University Medical Center, Nijmegen, The Netherlands; 2 Department of Obstetrics and Gynaecology, Canisius-Wilhelmina Hospital, Nijmegen, The Netherlands; 3 Department of Computing Sciences, Radboud University, Nijmegen, The Netherlands; 4 Department of Pathology, Radboud University Medical Center, Nijmegen, The Netherlands; 5 Department of Obstetrics and Gynecology, Haukeland University Hospital, Bergen, Norway; 6 Centre for Cancer Biomarkers, Department of Clinical Science, University of Bergen, Bergen, Norway; 7 Department for Health Evidence, Radboud University Medical Center, Nijmegen, the Netherlands; 8 Department of Pathology, Ghent University Hospital, Cancer Research Institute Ghent, Ghent, Belgium; 9 Department of Pathology and Molecular Genetics and Research Laboratory, Hospital Universitari Arnau de Vilanova, University of Lleida, IRBLleida, CIBERONC, Lleida, Spain; 10 Institute of Pathology, University Medical Center, Freiburg, Germany; 11 Department of Obstetrics and Gynecology, University Medical Center, Freiburg, Germany; 12 Institute of Veterinary Medicine, Georg-August-University, Goettingen, Germany; 13 Biomedical Research Group in Gynecology, Vall Hebron Institute of Research, Universitat Autònoma de Barcelona, CIBERONC, Barcelona, Spain; 14 Gynecological Department, Vall Hebron University Hospital, CIBERONC, Barcelona, Spain; 15 Pathology Department, Vall Hebron University Hospital, CIBERONC, Barcelona, Spain; 16 Department of Obstetrics and Gynecology, Hospital del Mar, PSMAR, Barcelona, Spain; 17 Mohn Medical Imaging and Visualization Centre, Department of Radiology, Haukeland University Hospital, Bergen, Norway; 18 Department of Pathology, University of Turku, Turku, Finland; 19 Obstetrics and Gynecology Department, Bichat-Claude Bernard Hospital, Paris, France; 20 Department of Gynecology and Obstetrics, University Hospital in Brno and Masaryk University, Brno, Czech Republic; 21 Department of Internal Medicine, Hematology and Oncology, University Hospital Brno and Masaryk University, Brno, Czech Republic; 22 Department of Pathology, University Hospital Brno and Masaryk University, Brno, Czech Republic; 23 Department of Pathology, Elisabeth-TweeSteden Hospital, Tilburg, The Netherlands; 24 Department of Oncology, KU Leuven, Leuven, Belgium; 25 Center for Gynecologic Oncology Amsterdam, Netherlands Cancer Institute and Amsterdam University Medical Center, The Netherlands; 26 Department of Pathology, Canisius-Wilhelmina Hospital, Nijmegen, The Netherlands; 27 Department of Data Science, University of Twente, Enschede, The Netherlands; University of Cambridge, UNITED KINGDOM

## Abstract

**Background:**

Bayesian networks (BNs) are machine-learning–based computational models that visualize causal relationships and provide insight into the processes underlying disease progression, closely resembling clinical decision-making. Preoperative identification of patients at risk for lymph node metastasis (LNM) is challenging in endometrial cancer, and although several biomarkers are related to LNM, none of them are incorporated in clinical practice. The aim of this study was to develop and externally validate a preoperative BN to predict LNM and outcome in endometrial cancer patients.

**Methods and findings:**

Within the European Network for Individualized Treatment of Endometrial Cancer (ENITEC), we performed a retrospective multicenter cohort study including 763 patients, median age 65 years (interquartile range [IQR] 58–71), surgically treated for endometrial cancer between February 1995 and August 2013 at one of the 10 participating European hospitals. A BN was developed using score-based machine learning in addition to expert knowledge. Our main outcome measures were LNM and 5-year disease-specific survival (DSS). Preoperative clinical, histopathological, and molecular biomarkers were included in the network. External validation was performed using 2 prospective study cohorts: the Molecular Markers in Treatment in Endometrial Cancer (MoMaTEC) study cohort, including 446 Norwegian patients, median age 64 years (IQR 59–74), treated between May 2001 and 2010; and the PIpelle Prospective ENDOmetrial carcinoma (PIPENDO) study cohort, including 384 Dutch patients, median age 66 years (IQR 60–73), treated between September 2011 and December 2013. A BN called ENDORISK (preoperative risk stratification in endometrial cancer) was developed including the following predictors: preoperative tumor grade; immunohistochemical expression of estrogen receptor (ER), progesterone receptor (PR), p53, and L1 cell adhesion molecule (L1CAM); cancer antigen 125 serum level; thrombocyte count; imaging results on lymphadenopathy; and cervical cytology. In the MoMaTEC cohort, the area under the curve (AUC) was 0.82 (95% confidence interval [CI] 0.76–0.88) for LNM and 0.82 (95% CI 0.77–0.87) for 5-year DSS. In the PIPENDO cohort, the AUC for 5-year DSS was 0.84 (95% CI 0.78–0.90). The network was well-calibrated. In the MoMaTEC cohort, 249 patients (55.8%) were classified with <5% risk of LNM, with a false-negative rate of 1.6%. A limitation of the study is the use of imputation to correct for missing predictor variables in the development cohort and the retrospective study design.

**Conclusions:**

In this study, we illustrated how BNs can be used for individualizing clinical decision-making in oncology by incorporating easily accessible and multimodal biomarkers. The network shows the complex interactions underlying the carcinogenetic process of endometrial cancer by its graphical representation. A prospective feasibility study will be needed prior to implementation in the clinic.

## Abstract 2

Casper Reijnen and co-workers report on risk prediction in endometrial cancer.

## Author summary

**Why was this study done?:**

- Bayesian networks are graphical networks that are based on machine learning and can be used for prediction purposes without the need to have values for all predictor variables available for each patient.
- Approximately 10% of patients with endometrial cancer have lymph node metastasis.
- The risk of lymph node metastasis and poor outcome differs substantially between individuals.
- Preoperative identification of patients at risk for lymph node metastasis and poor outcome allows tailoring of individualized treatment.

**What did the researched do and find?:**

- A Bayesian network to predict the risk of lymph node metastasis and survival was constructed with data from a retrospective multicenter development cohort from 10 centers across Europe (*n* = 763).
- The predictive capability of the final network was tested in 2 external cohorts from the Netherlands (PIpelle Prospective ENDOmetrial carcinoma [PIPENDO], *n* = 384) and from Norway (Molecular Markers in Treatment in Endometrial Cancer [MoMaTEC], *n* = 446).

**What do these findings mean?:**

- The Bayesian network we propose allows refined risk stratification before surgery and is easily usable.
- Because of its graphical character, the interactions between the different variables included into the network are directly visualized.
- A prospective feasibility study will be needed prior to implementation in the clinic.

## Introduction

In the era of personalized medicine, individualized treatment aims to minimize unnecessary exposure to therapy-related morbidity and at the same time offer proper management for high-risk patients. Bayesian networks (BNs) are graphical representations of probability distributions that visualize conditional probabilistic dependence relationships that often can be given a causal reading. These machine-learning–based computational models are well suited for prognostication and can be applied even when some patient findings are missing [1]. One advantage is that they enable to study the influence of all variables on one another, resembling clinical reasoning more closely than other models.

Although most patients with endometrial carcinoma (EC) present with early-stage disease and have a favorable prognosis, approximately 89,900 patients around the world died in 2018 as a consequence of this disease [2]. The presence of pelvic and/or para-aortic lymph node metastasis (LNM) is one of the most important prognostic factors for poor outcome. Identification of LNM during primary treatment allows patients to benefit from adequate adjuvant treatment because adjuvant treatment improves survival in node-positive EC [3,4]. However, routine lymphadenectomy in clinical early-stage EC has no impact on outcome and is associated with substantial long-term morbidity [5,6]. Consequently, no consensus exists regarding the selection of patients who will benefit from lymphadenectomy. The current guidelines are suitable to guide lymph-node–directed surgery based on clinicopathological factors [7]. Yet, approximately 50% of LNM is found in patients designated as low/intermediate risk, and a recent study showed that diagnostic accuracy for the prediction of LNM could be improved [8,9]. The introduction of sentinel-lymph–node (SLN) mapping provides a less invasive alternative strategy for evaluating lymph node status with only limited morbidity. However, this procedure can be particularly challenging in obese patients. Moreover, there is debate about the impact of non-SLN involvement in pelvic and para-aortic lymph nodes, supporting the need for noninvasive tools discriminating between patients with low risk and those with extensive nodal involvement [10].

The use of preoperative prediction models could decrease over- and undertreatment and allow prognosis-based shared decision-making. By identifying low-risk EC groups that do not need lymph-node–directed surgery, these tools could positively impact healthcare costs. Only a few models rely exclusively on preoperative data, none have been implemented into current guidelines, and, to our knowledge, only one performs with an area under the curve (AUC) above 0.75 for prediction of LNM, highlighting the need for more refined preoperative risk stratification [11–14]. The Cancer Genome Atlas (TCGA) has demonstrated the prognostic value of molecular classification in EC, which may be suitable for adjuvant treatment planning in the future [8,15,16]. So far, these subgroups have not been related to LNM in a preoperative setting nor to lymph-node–directed surgery. The p53-mutant subgroup was eminently identified as the subgroup with the poorest outcome and can be easily assessed by immunohistochemistry [17].

We sought to develop and externally validate a preoperative BN based on clinical, histopathological, and molecular biomarkers for the prediction of LNM and disease-specific survival (DSS) in EC patients.

## Methods

### Development cohort

A retrospective multicenter study was performed. The patients were identified from a previously published cohort and included patients treated between February 1995 and August 2013 for International Federation of Gynecology and Obstetrics (FIGO) stage I–IV endometrioid endometrial carcinoma (EEC) or nonendometrioid endometrial carcinoma (NEEC) at one of 10 participating European Network for Individualized Treatment of Endometrial Cancer (ENITEC) centers [16]. Participating centers were Radboud University Medical Center, Nijmegen, the Netherlands; University Medical Center Turku, Finland; KU Leuven, Belgium; University Hospital Brno, Czech Republic; University Medical Center Freiburg, Germany; Bichat-Claude Bernard Hospital, Paris, France; Haukeland University Hospital, Bergen, Norway; Hospital Universitari Arnau de Vilanova, Lleida, Spain; Vall Hebron University Hospital, Barcelona, Spain; and Hospital del Mar, Barcelona, Spain.

Only patients diagnosed by an expert gynecological pathologist, with complete clinical and pathological data and follow-up of at least 36 months were included, yielding a cohort of 1,199 patients. For the current study, preoperative endometrial biopsy slides were collected for assessment of the selected molecular biomarkers. This study was approved by the Institutional Review Board Radboud University Medical Center (Nijmegen, the Netherlands, Institutional Study Protocol 2015–2101). No informed consent was obtained because all data were analyzed anonymously. This study is reported as per the Strengthening the Reporting of Observational Studies in Epidemiology (STROBE) guideline (S1 STROBE Checklist).

### Selection and definition of variables

**Fig 1:**
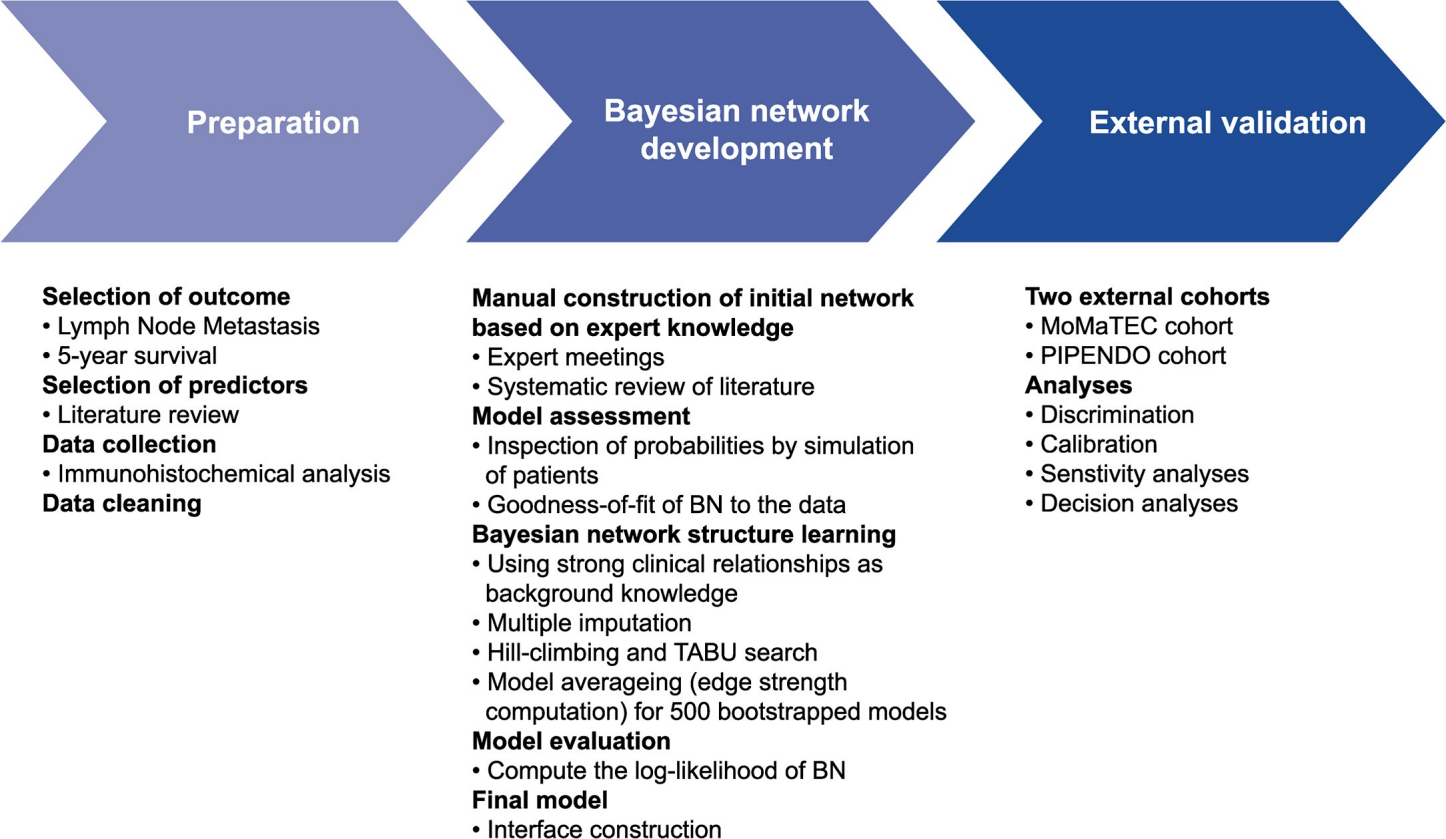
Summarizing overview of the BN development and validation. BN, Bayesian network; MoMaTEC, Markers for the Treatment of Endometrial Cancer; PIPENDO, PIpelle Prospective ENDOmetrial carcinoma.

An overview of study procedures can be appreciated from Fig 1. This plan was constructed before the study had begun to produce data. Potential preoperative clinical and histopathological variables with prognostic value for the prediction of LNM were identified by a systematic review of the literature (Table 1) [18]. Four validated molecular biomarkers were selected for immunohistochemical staining on preoperative biopsy samples: estrogen receptor (ER), progesterone receptor (PR), L1 cell adhesion molecule (L1CAM), and p53. Loss of ER and PR was validated as independent prognostic markers for the prediction of LNM [8]. From a biological perspective, loss of ER was associated with epithelial-to-mesenchymal transition (EMT) [19]. L1CAM was validated as a strong prognostic biomarker in EC and was also associated with EMT [16,20]. Research by TCGA has shown that p53 status identified patients who had an inherent poor prognosis [15]. Adjuvant therapy was given according to regional or national protocols. Outcome variables were the presence of pelvic and/or para-aortic LNM, disease recurrence, and DSS at 1, 3, and 5 years. Disease recurrence was classified as local (vaginal vault), regional (involving pelvic structures), or distant (other recurrences).

**Table 1:** Candidate variables for the construction of the BN.

Variables	Cutoff value(s)
**Preoperative clinical and histopathological variables**	
Age	<70; ≥70 years
Body mass index	<25; ≥25 kg/m^2^
Hemoglobin*	<12; ≥12 g/dl
Leukocyte counts*	≤10 × 10^9^; >10 × 10^9^/l
Thrombocyte counts*	<400 × 10^9^; ≥400 × 10^9^/l
Ca-125 serum levels*	<35; ≥35 IU/ml
Lymphadenopathy on MRI or CT	No; yes (≥10 mm short axis diameter)
Cervical cytology	No; yes (atypical endometrial cells present)
Tumor grade†	1; 2; 3
Tumor histology†	Endometrioid; non-endometrioid
**Preoperative molecular biomarkers**	
ER expression	<10; ≥10% of tumor cells with nuclear staining
PR expression	<10; ≥10% of tumor cells with nuclear staining
L1CAM expression	<10; ≥10% of tumor cells with membranous staining
p53 expression	Wild type; aberrant‡
**Postoperative variables**	
Myometrial invasion	No invasion; <50%; ≥50%
Lymphovascular space invasion	No; yes
Cervical invasion	No; yes
FIGO stage	IA; IB; II; IIIA; IIIB; IIIC; IV
Tumor grade§	1; 2; 3¶
Tumor histological subtype§	Endometrioid; nonendometrioid
Adjuvant therapy	None; radiotherapy; chemotherapy; chemoradiation; other

*Preoperative serum levels, assessed within 2 weeks before surgery.

†Tumor grade and histology assessed in preoperative endometrial biopsy.

‡Expression was considered aberrant when nuclear staining was completely absent or when more than 80% of tumor cell nuclei exhibited strong expression.

§Tumor grade and histological subtype assessed in hysterectomy specimens.

¶Grade 3 includes grade 3 EECs and NEECs.

**Abbreviations:** BN, Bayesian network; Ca-125, cancer antigen 125; CT, computed tomography; EC, endometrial carcinoma; EEC, endometrioid EC; ER, estrogen receptor; FIGO, International Federation of Gynecology and Obstetrics; L1CAM, L1 cell adhesion molecule; MRI, magnetic resonance imaging; NEEC, non-endometrioid EC; PR, progesterone receptor.

### Immunohistochemical analysis of endometrial biopsies

Detailed information on tissue processing, immunohistochemical analysis, and scoring can be found in S1 Text. ER and PR antibodies were generously provided by Dako (Agilent Technologies, Santa Clara, CA, USA). Scoring of immunohistochemical staining was performed twice by assessors blinded to pathological and clinical characteristics (N.V., H.K., J.B., K.v.d.V., C.R.). Discrepancies in scoring were reviewed in a consensus meeting with all assessors.

### Bayesian network development

The BN was developed through an iterative process, including stepwise construction of different network structures using manual construction and a data-driven approach. First, manual construction was performed because the use of expert knowledge is known to improve structure learning processes [21]. Based on systematic review and expert meetings, potential preoperative predictors were selected. These variables were displayed by the BN as nodes, and causal relationships were defined and visualized as arrows connecting the nodes. As a starting point for the network structure, all variables important for creating a causal network representative of natural tumor progression were included: postoperative tumor grade, myometrial invasion (MI), and lymphovascular space invasion (LVSI). These variables were, however, not used for prediction of the outcomes. Subsequently, the selected preoperative predictors were added to the structure. A likelihood was calculated to determine after each cycle how well the network fitted the data set. A higher log-likelihood indicates that a model explains a given data set more accurately.

Second, a data-driven approach using score-based machine learning algorithms was adopted to further improve the BN. Specifically, the hill-climbing and Tabu-search algorithms were used. Bootstrap resampling was applied to learn 500 network structures. Arc strengths (between 0 and 1) were computed, including the frequency of an edge (approximating its clinical significance) between 2 variables across the 500 bootstrap-resampled network structures. Edges with arc strength > 0.7 were considered for inclusion in the BN. Again, log-likelihood was used to establish the goodness of fit to the data of the resulting BN model. Multiple imputation was employed to impute missing data. This method calculated missing values by using all the nodes of the BN as evidence in 500 random samples, which were averaged for each new observation. The network was developed and imputation was performed using R (3.3.2) with the bnlearn package (4.4.1).

### BN validation

Validation was performed by using only preoperative variables as predictor variables. Network performance was assessed based on overall performance, calibration, and discrimination testing. The model’s overall performance was quantified by the Brier score, which is the mean squared difference between each predicted probability and the observed outcome, between 0 and 1. A lower Brier score indicates better accuracy of the probabilistic predictions. Discrimination was assessed using a receiver operating characteristic (ROC) curve generated by plotting sensitivity against 1-specificity. Discriminative performance was quantified based on the AUC. Calibration was visualized using calibration plots, in which the predicted outcome was plotted against the observed outcome. To quantify model calibration, the predicted number of events was compared with the observed number. Sensitivity analyses were performed by omitting molecular markers and clinical markers, respectively. Finally, decision analysis curves were plotted to assess the net benefit of BN-assisted decisions.

Performance was validated using 2 prospective multicenter external data sets: the Molecular Markers in Treatment in Endometrial Cancer (MoMaTEC) cohort (*n* = 446), including patients treated between May 2001 and 2010, and the PIpelle Prospective ENDOmetrial carcinoma (PIPENDO) cohort (*n* = 384), including patients treated between September 2011 and December 2013 [8,22]. Patients from these cohorts were only included if they had a minimal subset of variables: preoperative tumor grade, at least 3 molecular biomarkers, at least one of the clinical preoperative markers, and information on outcome. The MoMaTEC cohort was used to validate both outcomes (LNM and 5-year DSS) because all included patients had undergone lymphadenectomy; the PIPENDO cohort was used to validate 5-year DSS only because information on LNM was only available for a subset of patients. Analyses were performed using IBM SPSS version 25.0 (SPSS IBM, New York, NY, USA) and R (3.3.2), with the caTools (1.17.1.2), pROC (1.14.0) and caret packages (6.0–84).

## Results

### Patients

Preoperative endometrial biopsies were available from 809 of the 1,199 patients (67.4%) in the development cohort. Subsequently, 46 patients (3.8%) were excluded because of insufficient tumor tissue in their endometrial biopsy, leaving 763 patients for analysis (S1 Fig). Baseline characteristics are shown in Table 2 and did not differ significantly from those of excluded patients, except for cervical invasion (*P* = 0.03, S1 Table). The number of missing data for which imputation was used can be appreciated from Table 2 as well. A total of 215 cases had information on all predictor variables. The MoMaTEC validation cohort consisted of 446 patients, and the PIPENDO validation cohort of 384 patients (Table 2).

**Table 2:** Baseline characteristics of development cohort and 2 validation cohorts.

Variable	Development cohort	Validation cohort MoMaTEC	Validation cohort PIPENDO
	(*N* = 763)	(*N* = 446)	(*N* = 384)
Age (y)	65 (58–71)	64 (59–74)	66 (60–73)
BMI (kg/m^2^)	29 (26–33)	27 (24–32)	29 (25–33)
Follow-up (months)	60 (45–74)	54 (28–71)	50 (33–59)
Tumor grade, preoperative			
—1	372 (48.8)		182 (47.4)
—2	173 (22.7)	374 (83.9)*	99 (25.8)
—3	110 (14.4)	72 (16.1)	103 (26.8)
—Unknown	108 (14.2)		
ER expression			
—Positive	686 (89.9)	345 (77.4)	342 (89.1)
—Negative	76 (10.0)	101 (22.6)	41 (10.7)
—Unknown	1 (0.1)		1 (0.3)
PR expression			
—Positive	620 (81.3)	335 (75.1)	298 (77.6)
—Negative	137 (18.0)	109 (24.4)	85 (22.1)
—Unknown	6 (0.8)	2 (0.4)	1 (0.3)
L1CAM-expression			
—Negative	665 (87.2)	396 (88.8)	328 (85.4)
—Positive	79 (10.4)	32 (7.2)	55 (14.3)
—Unknown	19 (2.5)	18 (4.0)	1 (0.3)
p53 expression			
—Wild type	584 (76.5)	218 (48.9)	275 (71.6)
—Mutant	112 (14.7)	66 (14.8)	107 (27.9)
—Unknown	67 (8.8)	162 (36.3)	2 (0.5)
Ca-125			
—≤35 IU/ml	318 (41.7)	41 (9.2)	221 (57.6)
—>35 IU/ml	90 (11.8)	14 (3.1)	89 (23.2)
—Unknown	355 (46.5)	391 (87.7)	74 (19.3)
Thrombocytes			
—<400 × 10^9^/l	557 (73.0)	249 (55.8)	146 (38.0)
—≥400 × 10^9^/l	25 (3.3)	29 (6.5)	18 (4.7)
—Unknown	181 (23.7)	168 (37.7)	220 (57.3)
Imaging results			
—No lymphadenopathy	460 (60.3)	110 (24.7)	160 (41.7)
—Lymphadenopathy	38 (5.0)	17 (3.8)	26 (6.8)
—Unknown	265 (34.7)	319 (71.5)	198 (51.6)
Cervical cytology			
—Normal	406 (53.2)	127 (28.5)	285 (74.2)
—Abnormal	27 (3.5)	62 (13.9)	37 (9.6)
—Unknown	330 (43.3)	257 (57.6)	62 (16.1)
Tumor grade†			
—1	317 (41.5)	171 (38.3)	140 (36.5)
—2	289 (37.9)	142 (31.8)	127 (33.1)
—3	157 (20.6)	133 (29.8)	117 (30.5)
Histological subtype†			
—EEC	714 (93.6)	367 (82.3)	307 (79.9)
—NEEC	49 (6.4)	79 (17.7)	77 (20.1)
—Unknown		1 (0.2)	
MI			
—<50%	477 (62.5)	273 (61.2)	208 (54.2)
—≥50%	283 (37.1)	172 (38.8)	176 (45.8)
—Unknown	3 (0.4)		
Cervical invasion			
—No	591 (77.5)	371 (83.2)	340 (88.5)
—Yes	86 (11.3)	71 (15.9)	44 (11.5)
—Unknown	86 (11.3)	4 (0.9)	
FIGO stage			
—IA	428 (56.1)	243 (54.5)	197 (51.3)
—IB	196 (25.7)	102 (22.9)	125 (32.6)
—II	51 (6.7)	38 (8.5)	23 (6.0)
—IIIA	20 (2.6)	6 (1.3)	11 (2.9)
—IIIB	4 (0.5)	1 (0.2)	4 (1.0)
—IIIC	43 (5.6)	43 (9.6)	17 (4.4)
—IV	19 (2.5)	12 (2.7)	7 (1.8)
LVSI			
—No	435 (57.0)	91 (20.4)	80 (72.9)
—Yes	96 (12.6)	43 (9.6)	104 (27.1)
—Unknown	232 (30.4)	312 (70.0)	
Lymph nodes			
—Negative	440 (57.7)	394 (88.3)	57 (14.8)
—Positive	53 (6.9)	52 (11.7)	19 (4.9)
—Unknown	270 (35.4)		308 (80.2)
Treatment			
—None	415 (54.4)	323 (72.4)	206 (53.6)
—Radiotherapy	283 (37.1)	43 (9.6)	142 (37.0)
—Chemotherapy	38 (5.0)	77 (17.3)	33 (8.6)
—Chemoradiation	26 (3.4)	1 (0.2)	3 (0.8)
—Hormonal	0 (0)	2 (0.4)	0 (0)
—Unknown	1 (0.1)		

Continuous variables are presented as median (with IQR).

*Grade 1 and 2 combined.

†Tumor grade and histology assessed in hysterectomy specimen.

**Abbreviations:** BMI, body mass index; Ca-125, cancer antigen 125; EEC, endometrioid endometrial carcinoma; ER, estrogen receptor; FIGO, international federation of gynecology and obstetrics; L1CAM, L1 cell adhesion molecule; LVSI, lymphovascular space invasion; MI, myometrial invasion; MoMaTEC, Markers for the Treatment of Endometrial Cancer; NEEC, non-endometrioid endometrial carcinoma; PIPENDO, PIpelle Prospective ENDOmetrial carcinoma; PR, progesterone receptor.

### BN development

**Fig 2:**
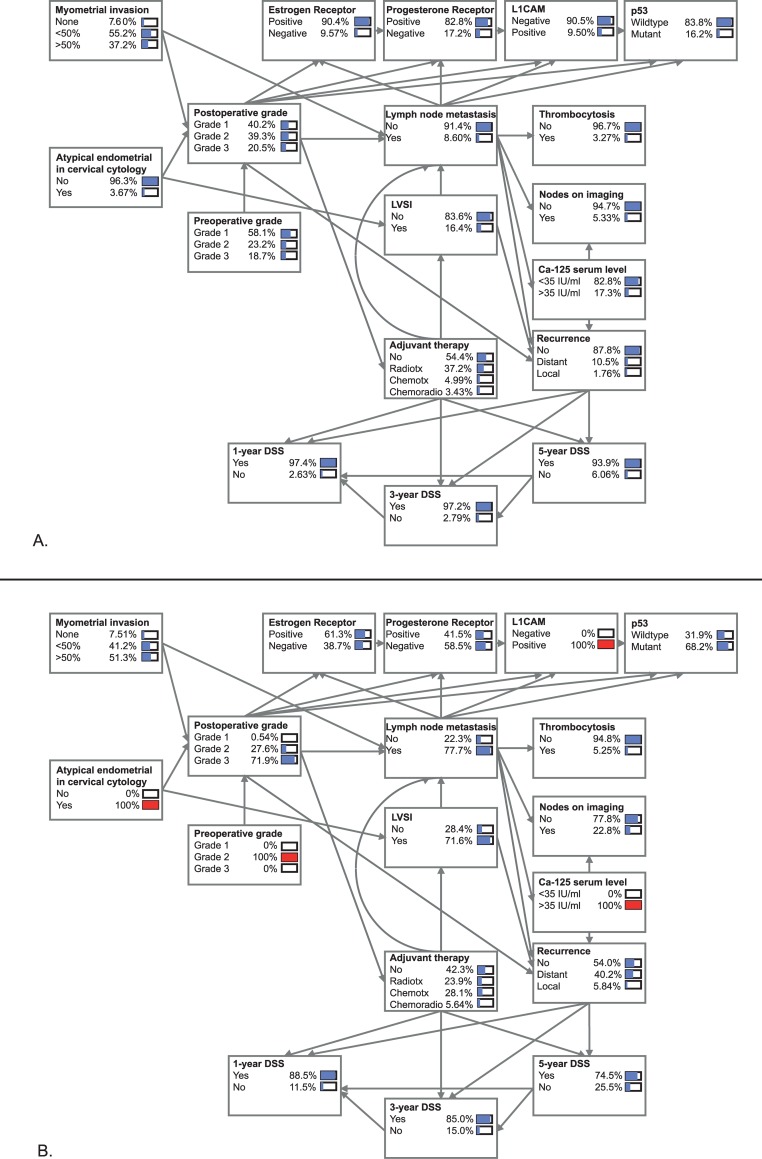
Final BN for the prediction of LNM and 5-year DSS. (A.) Probability estimates are shown when no markers were recorded. (B.) Example of probability estimates in a case with preoperative tumor grade (grade 2), cervical cytology (atypical endometrial cells present), L1CAM expression (positive), and Ca-125 serum levels (>35 IU/ml). Probability distributions are shown in the nodes, and dependencies are indicated by the arrows connecting the nodes. If variables are not connected directly or indirectly, they are assumed to be (conditionally) independent. Often, the direction of the arrows can be given causal meaning. Red bars indicate that the specific variable is instantiated, i.e., a specific value or evidence is provided. Blue bars in the bar plots indicate the resulting probabilities of the probability distributions. Because of imputation, probability distributions vary slightly from Table 2. BN, Bayesian network; Ca-125, cancer antigen 125; DSS, disease-specific survival; LNM, lymph node metastasis; LVSI, lymphovascular space invasion; L1CAM, L1 cell adhesion molecule.

The final BN is depicted in Fig 2. We elected not to include age, BMI, comorbidity, histology, hemoglobin, or leukocytes because the arc strengths obtained from machine learning were <0.7, which was supported by recent systematic reviewing of literature showing only moderate performance [18]. According to BN construction, all variables that represent natural tumor progression were included, e.g., LVSI and MI, yet only preoperative variables were used for prognostication. All probability distributions are shown in the nodes, and dependencies are indicated by the arrows connecting the nodes. Variables linked to each other are assumed to be dependent. If variables are not connected directly or indirectly, they are assumed to be independent. Also, the direction of the arrows represents causality; e.g., “myometrial invasion → lymph node metastasis” can be read as “myometrial invasion causes lymph node metastasis.” Multiple arrows pointing toward the same variable indicate that the variable is the consequence of more than one cause.; e.g., “myometrial invasion → lymph node metastasis” and “LVSI → lymph node metastasis” represent 2 separate but interacting causes of LNM.

### Application of the BN

Probability distributions without input from the other variables are represented in Fig 2A. If the network is provided with evidence from patient findings, the probability distributions are automatically updated; e.g., if information about preoperative tumor grade (grade 2), L1CAM-expression (positive), cervical cytology (atypical endometrial cells present), and cancer antigen 125 (Ca-125) level (elevated) is added, the probability of having LNM increases from 8.6% to 77.7% (Fig 2B). Also, probability estimates of all other variables included in the network can be extracted; e.g., in this specific situation, the probability of a grade 3 tumor in the hysterectomy specimens increases from 18.7% to 73.9%, and the probability of LVSI increases from 16.4% to 71.6%.

**Fig 3:**
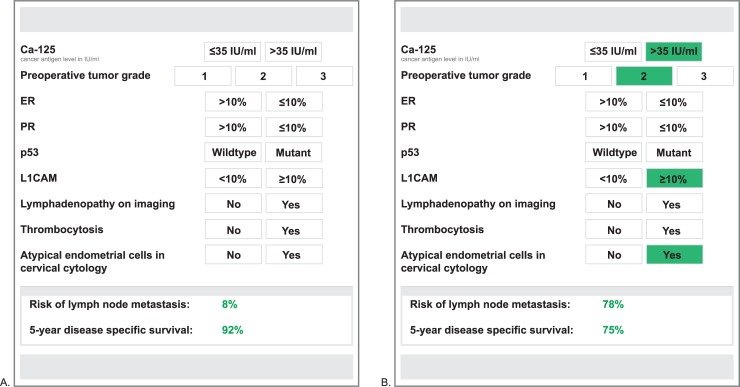
Concept web-based interface of the BN. The baseline probability estimates for LNM and 5-year DSS (visualized in panel A) are interactively updated when variables are provided to the model (visualized in panel B). BN, Bayesian network; Ca-125; cancer antigen 125; DSS, disease-specific survival; ER, estrogen receptor; LNM, lymph node metastasis; L1CAM, L1 cell adhesion molecule; PR, progesterone receptor.

To further explain the BN’s behavior, S2 Table provides examples of the probability estimates for LNM in different situations. We developed an app-based tool using the BN to estimate the probability of LNM and 5-year DSS in individual patients that will be treated for EC (concept is shown in Fig 3).

### Validation

**Fig 4:**
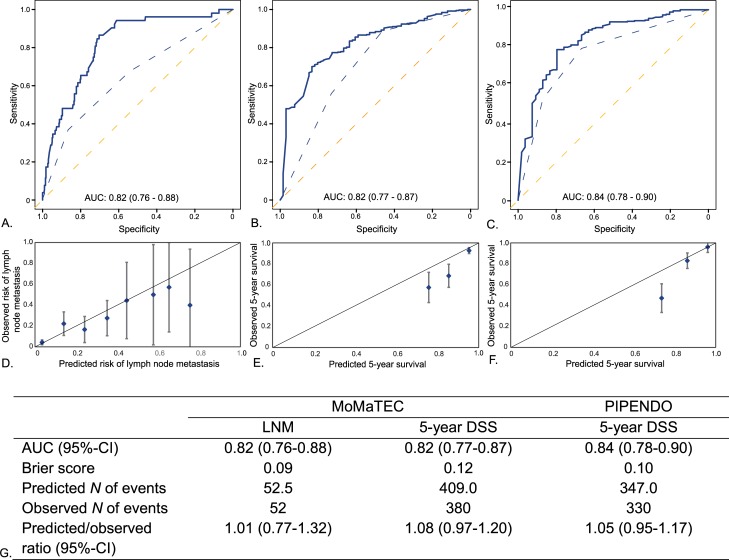
ROC curves. (A) Prediction of LNM in the MoMaTEC cohort, (B) prediction of 5-year DSS in the MoMaTEC cohort, and (C) prediction of 5-year DSS in the PIPENDO cohort. Calibration plots for (D) prediction of LNM in the MoMaTEC cohort, (E) prediction of 5-year DSS in the MoMaTEC cohort, and (F) prediction of 5-year DSS in the PIPENDO cohort. (G) Concordance statistics of the BN. The solid blue lines represent the ROC curves obtained by ENDORISK. The blue dotted lines represent the ROC curves including obtained by using only preoperative tumor grade as predictor (as a reference). The vertical bars in panel D represent 95% CIs. AUC, area under the curve; BN, Bayesian network; CI, confidence interval; DSS, disease-specific survival; ENDORISK, preoperative risk stratification in endometrial cancer; LNM, lymph node metastasis; MoMaTEC, Markers for the Treatment of Endometrial Cancer; PIPENDO, PIpelle Prospective ENDOmetrial carcinoma; ROC, receiver operating characteristic.

Validation was performed by using only the preoperative variables as predictor variables. The AUC was 0.82 (95% confidence interval [CI] 0.76–0.88) for LNM and 0.82 (95% CI 0.77–0.87) for 5-year DSS in the MoMaTEC cohort (Fig 4). Fig 4 depicts ROC curves compared to ROC curves obtained from using only classic histopathological markers (tumor grade) as predictor variables. The Brier scores were 0.09 for LNM and 0.12 for 5-year DSS, respectively. For 5-year DSS in the PIPENDO cohort, the AUC was 0.84 (95% CI 0.78 to 0.90), and the Brier score was 0.10.

The prediction of LNM was well-calibrated with the observed LNM for the external MoMaTEC cohort (Fig 4). The prediction of 5-year DSS was well-calibrated with the observed 5-year DSS for both external data sets, with a trend toward overestimating survival in the lower predicted survival rates. The ratio of predicted/observed cases for LNM was 1.01 (95% CI 0.77–1.32) in the MoMaTEC cohort. The ratio of predicted/observed cases for 5-year DSS was 1.08 (95% CI 0.97 to 1.20) and 1.05 (95% CI 0.95 to 1.17) in the MoMaTEC and PIPENDO cohorts, respectively.

The number of positive predictions for different cutoff values is shown in S3 Table, as well as sensitivity, specificity, positive predictive values (PPVs), and negative predictive values (NPVs). With the BN, 249 patients (55.8%) were classified as having a risk of LNM <5%, with a false-negative rate of 1.6%. The false-positive rate for this cutoff was 76%. The network’s predictions in the MoMaTEC cohort were then categorized into risk groups based on observed prevalence of LNM (Table 3). Groups were identified as carrying a very low (0%), low (1.8%), intermediate (17%), high-intermediate (21%), and high (36%) risk of LNM.

**Table 3:** Risk groups assigned based on predicted probabilities by the ENDORISK BN.

Predicted probability	Risk group	Observed prevalence LNM in risk group	*N* of patients assigned to risk group
<1%	Very low	0/24 (0%)	24/446 (5.4%)
1%–5%	Low	4/225 (1.8%)	225/446 (50%)
6%–15%	Intermediate	14/84 (17%)	84/446 (19%)
16%–25%	High-intermediate	9/43 (21%)	43/446 (10%)
>25%	High	25/70 (36%)	70/446 (16%)

**Abbreviations:** BN, Bayesian network; ENDORISK, preoperative risk stratification in endometrial cancer; LNM, lymph node metastasis.

To investigate the impact of different predictors, sensitivity analyses were performed, including only clinical and histological biomarkers (Ca-125, lymphadenopathy on imaging, cervical cytology, thrombocytosis, and preoperative tumor grade) and molecular and histological biomarkers (ER, PR, L1CAM, p53, and preoperative tumor grade), showing negative impact on discrimination metrics (S4 Table).

Because AUC analysis and calibration plots are unable to evaluate whether prediction models improve clinical decision-making, decision analysis curves were constructed. The net benefit using preoperative risk stratification in endometrial cancer (ENDORISK) (red lines), the net benefit made with the assumption that none has the outcome of interest (black lines), and the net benefit made with the assumption that all have the outcome of interest (gray line) are shown in S2 Fig. Use of the BN predictions for LNM to inform clinical decisions was better than a scenario in which all patients were treated or in which no patient was treated, with a risk probability ranging between 0.05 and 0.55.

## Discussion

We have developed and externally validated the ENDORISK BN for EC patients, based on molecular, histological, and clinical biomarkers, to predict LNM and 5-year DSS. External validation revealed high discriminative performance and good calibration for both outcomes. ENDORISK was able to classify 55.8% of the patients as at <5% risk for LNM, with a false-negative rate of 1.6%.

In the era of “personalized medicine,” the use of prediction models has gained increasing interest among clinicians to guide treatment planning. Routine lymphadenectomy in clinical early-stage EC has not been demonstrated to improve outcome and is associated with surgery-related morbidity of 15% to 20% [6]. However, selective surgery to identify those patients with LNM in a primary setting is crucial because 5-year survival is 60% to 65% after adjuvant therapy for LNM [3]. The ENDORISK model is based on variables that can be assessed preoperatively, and it could therefore support patient counseling and shared decision-making before surgery. This model informs both the clinician and patient with individualized risk estimates weighed against patients’ preferences and the extent of the surgical approach. Instead of providing risk groups, this model presents individualized and continuous risk estimates, with the potential to improve tailored treatment planning. More specifically, ENDORISK could help to decide whether to perform lymph-node–directed surgery or support physicians in deciding whether to perform lymphadenectomy when a side-specific SLN cannot be detected. Using the model allows identification of a low-risk (<5%) subgroup, with a false-negative rate of 1.6%, which may lead to selective omission of lymph-node–directed surgery. Acceptable cutoff values have to be weighed against patients’ preferences, comorbidity, and age. We have shown that different risk groups can be identified, which could be used to support shared decision-making in clinical practice. Before implementation in the clinic, prospective evaluation will be necessary. The mobile application can be easily used in outpatient clinic settings to inform clinicians and patients during clinical decision-making. In this way, ENDORISK can interactively be used requiring not more than a few minutes. Further implementation may be facilitated by the development of a decision aid tool.

Few attempts have been made to predict the risk of LNM before surgical treatment [11–13]. Koskas and colleagues evaluated the performance of these models within their cohort of 519 patients [11–14]. Only one model had an AUC > 0.75, highlighting the need for improved preoperative risk stratification [11]. Integration of molecular classification of EC could improve risk stratification by providing robust biomarkers that are more reflective of actual tumor biology [15]. The “copy-number high” subgroup was eminently identified as the subgroup with the poorest prognosis and is reflected by abnormal immunohistochemical p53 expression. Anticipating on the rise of molecular classification this biomarker was included into the BN. Preoperative ER and PR expression, evaluated in the prospective MoMaTEC1-trial, predict LNM with an adjusted odds ratio of 2.0 [8]. L1CAM expression, validated as a preoperative prognostic biomarker, predicts LNM with an adjusted odds ratio of 2.5 and was shown to refine the “p53wt” subgroup [16,23].

Our model incorporated both molecular and clinical information and exhibited high diagnostic performance in 2 rounds of external validation. Because BNs determine causes and effects based on the conditional probabilities between variables, they can be used to make meaningful predictions even when not all variables are available, in contrast to traditional regression models that require all predictor variables to be known. Moreover, the visual network structure provides interpretable predictions on all variables and allows clinical reasoning based on intuitive connections between variables in medical data [1].

In an attempt to decrease morbidity associated with lymphadenectomy, a shift is taking place toward less invasive SLN mapping, which allows evaluation of SLN with only limited morbidity [24]. Identification of the SLN is successful in 81% of patients, which has led to an algorithm that prescribed side-specific lymphadenectomy in the event that SLN cannot be detected in a hemipelvis [24,25]. With the ENDORISK model, we correctly identified 55% of patients with extremely low risk of LNM, in whom lymph node evaluation might be omitted in specific cases such as morbidly obese or fragile patients [10]. The exact role of SLN mapping in high-risk patients, especially with nonendometrioid histology, remains to be elucidated [26]. Patients with LNM could benefit from surgical removal of metastatic lymph nodes, as a retrospective study including 12,333 patients showed that the extent of nodal resection significantly was associated with improved survival in node-positive patients (53% in patients with fewer than 10 nodes removed to 72% in those with more than 20 nodes removed) [27]. To note, this study was unsuited to draw any conclusions on causality. Moreover, uncertainty of para-aortic node status may result in inappropriate adjuvant therapy choices, including noninclusive radiation fields.

The current study has some limitations. Inherent to the retrospective nature of the study, missing values were present, and techniques were used to impute the missing data. For the external validation, imputation was not applied because the nature of BNs allows for missing predictor variables. Moreover, the clinical applicability of ENDORISK to rare histological subtypes of ECs, e.g., clear-cell carcinomas or undifferentiated carcinomas, remains uncertain because these cases constitute only a small subgroup within our cohort. We have chosen to include p53 expression in the ENDORISK model because abnormal p53 was eminently identified as the subgroup with the poorest prognosis, and p53 status can be easily assessed with immunohistochemistry [17]. We have chosen to include immunohistochemical biomarkers to have this model easily and widespread incorporated in clinical practice.

Dynamic prediction modeling is essential to allow a model to stay up to date with changing treatments and new biomarkers. One of the advantages of BNs is that they can be updated with new evidence, allowing them to evolve over time through the incorporation of new data. They can be updated with new data from variables already included in the BN but also with information on new candidate biomarkers. With the increasing availability of molecular techniques, the inclusion of these immunohistochemical biomarkers is the first step anticipating on adding molecular information such as *polymerase-ε* (*POLE*) and microsatellite instability (MSI) status in the future if value is demonstrated in the preoperative setting. Moreover, high-potential imaging biomarkers could include expert ultrasound and novel structural and functional imaging techniques by MRI.

This study illustrates how BNs can be used for individualizing clinical decision-making in oncology by incorporating easily accessible and multimodal biomarkers. We developed the preoperative ENDORISK BN model for patients with EC to predict LNM and 5-year DSS, using molecular, histological, and clinical biomarkers. External validation revealed high diagnostic performance. The network improves understanding of the complex interactions underlying the carcinogenetic process of endometrial cancer by its graphical representation. By applying ENDORISK in a representative endometrial cancer cohort, 55.8% of patients were classified as low risk, with a false-negative rate of 1.6% (4/249). To investigate whether ENDORISK positively impacts individualized treatment, prospective evaluation is necessary incorporating patient-reported outcome measures.

### URL

The ENDORISK BN can be downloaded from http://www.cs.ru.nl/~peterl/endomcancer.html.

## Supporting information

S1 STROBE checklist STROBE reporting checklist. STROBE, Strengthening the Reporting of Observational Studies in Epidemiology.
(DOCX)

S1 Text Detailed information on immunohistochemical staining. (PDF)

S1 Fig Study flowchart. (PDF)

S2 Fig **Decision curves for (A) LNM in the MoMaTEC cohort, (B) 5-year DSS in the MoMaTEC cohort, and (C) 5-year DSS in the PIPENDO cohort.** DSS, disease-specific survival; LNM, lymph node metastasis; MoMaTEC, Molecular Markers in Treatment in Endometrial Cancer; PIPENDO; PIpelle Prospective ENDOmetrial carcinoma.
(PDF)

S1 Table Baseline characteristics of included versus excluded patients. (PDF)

S2 Table Probability estimates for LNM given different evidence situations. LNM, lymph node metastasis.
(PDF)

S3 Table Diagnostic accuracy values for the prediction of LNM in the MoMaTEC cohort, using various cutoff values. LNM, lymph node metastasis; MoMaTEC, Molecular Markers in Treatment in Endometrial Cancer.
(PDF)

S4 Table Sensitivity analysis for external validation, omitting clinical variables and molecular markers. (PDF)

## Abbreviations

AUC: area under the curve
BN: Bayesian network
Ca-125: cancer antigen 125
CI: confidence interval
CT: computed tomography
DSS: disease-specific survival
EC: endometrial carcinoma
EEC: endometrioid EC
EMT: epithelial-to-mesenchymal transition
ENDORISK: preoperative risk stratification in endometrial cancer
ENITEC: European Network for Individualized Treatment of Endometrial Cancer
ER: estrogen receptor
FIGO: International Federation of Gynecology and Obstetrics
IQR: interquartile range
LNM: ymph node metastasis
LVSI: lymphovascular space invasion
L1CAM: L1 cell adhesion molecule
MI: myometrial invasion
MoMaTEC: Molecular Markers in Treatment in Endometrial Cancer
MRI: magnetic resonance imaging
MSI: microsatellite instability
NEEC: non-endometrioid EC
NPV: negative predictive value
PIPENDO: PIpelle Prospective ENDOmetrial carcinoma
*POLE*: *polymerase-ε*
PPV: positive predictive value
PR: progesterone receptor
ROC: receiver operating characteristic
SLN: sentinel lymph node
STROBE: Strengthening the Reporting of Observational Studies in Epidemiology
TCGA: The Cancer Genome Atlas
