# Supplementary material for: Preoperative risk stratification in endometrial cancer (ENDORISK) by a Bayesian network model: A development and validation study
Source: PLoS Med. 2020 May 15;17(5):e1003111. doi: 10.1371/journal.pmed.1003111 (PMC7228042; doi:10.1371/journal.pmed.1003111)
Supplement: S1 Text — (PDF) [file pmed.1003111.s002.pdf]

S1 Text. Detailed information on immunohistochemical staining.

Immunohistochemical staining

For ER and PR, antigen retrieval (97 ^{0}C for 30 minutes in Tris/EDTA buffer pH 9 [Envision FLEX Target Retrieval Solution High pH, DAKO, Agilent Technologies, Santa Clara, CA, United States]) and blocking of endogenous peroxidase with hydrogen peroxide were performed. Subsequently, slides were incubated with: ER antibody (clone EP1 GA084, DAKO, Agilent Technologies, Santa Clara, CA, United States) and PR antibody (clone, Pgr 1294 GA090, DAKO, Agilent Technologies, Santa Clara, CA, United States). Envision FLEX/HRP (DAKO, Agilent Technologies, Santa Clara, CA, United States) was used and visualization was performed using Envision FLEX DAB+ Chromogen (DAKO, Agilent Technologies, Santa Clara, CA, United States).

For L1CAM, EDTA (95 ^{0}C for 10 minutes in Tris-EDTA buffer pH 9) and blocking of endogenous peroxidase with hydrogen peroxide were performed. Subsequently, slides were incubated with: L1CAM antibody (purified anti-CD171, clone 14.10, Biolegend, San Diego, CA, US, dilution 1:100). Powervision+ Poly-HRP was used and visualization was performed using PowerVision DAB substrate solution (Leica Biosystems, Buffalo Grove, IL, US).

For p53 staining, antigen retrieval (30 minutes, pH 6·7) and blocking of endogenous peroxidase with hydrogen peroxide were performed. Subsequently, slides were incubated with p53 antibody (clone DO-7 + BP53-12, dilution 1:600). Powervision+ Poly-HRP was used and visualization was performed using PowerVision DAB substrate solution (Leica Biosystems, Buffalo Grove, IL, US). Counterstaining was performed with hematoxylin, and slides were dehydrated and mounted.

For the subgroup of patients from the Haukeland university hospital, Bergen, Norway (166 patients), staining protocol differed slighty from the abovementioned staining protocols. In short, tissue microarrays (TMAs) were constructed for all endometrial biopsies with three

tissue cylinders from each case. Microwave antigen retrieval (750 W for 10 and 350 W for 15 min) in Tris–EDTA buffer pH 9 was performed before peroxidase blocking (Dako S-2032) for 5 min. and incubation with: Oestrogen Receptor α (ER) (Dako M7047) diluted 1:50, Progesterone Receptor (PR) (Dako M3569) diluted 1:150 both for 30 min, purified anti-CD171 antibody clone 14.10 (Biolegend, San Diego, CA, US) diluted 1:100; and tumour protein 53 (p53) (Dako M7001) diluted 1:1000 for 60 min. Subsequently, the EnVision+Mouse HRP labelled polymer secondary antibody with DAB+ (K4006) was used. Slides were counterstained with Dako Automation Haematoxylin.

Scoring of immunohistochemistry

For ER and PR, the number of stained tumor nuclei was scored, and subsequently cases were dichotomized, using 10% as a cut-off value. For L1CAM, the number of tumor cells showing membranous expression was scored and cases were dichotomized, using 10% as a cut-off value. For p53, staining was considered abnormal when there was complete absence of nuclear staining (null-expression) or when more than 80% of tumor cell nuclei showed strong expression (overexpression).

For the Bergen subgroup, scoring was performed at the Haukeland university hospital and differed slighty from the Radboud staining protocols. In short, both intensity and area of positive tumour cells were scored. The intensity was scored from 0 (no staining) to 3 (strong), and the area as 0, 1 (<10%), 2 (10–50%) and 3 (51–100%). From this, a staining index (0–9) was calculated as the product of intensity and area. If heterogeneity was seen for the three tissue cylinders of each case, the three cylinders were given one overall averaged score. For L1CAM staining, index was dichotomized using ≥ 4 as a cut-off for positive expression. For ER staining index ⩽3 and PR staining index 0 was defined as negative expression. Pathologic expression of p53 (high) was defined as staining index ⩾4.
