# Supplementary figures and images for: Preoperative risk stratification in endometrial cancer (ENDORISK) by a Bayesian network model: A development and validation study
Source: PLoS Med. 2020 May 15;17(5):e1003111. doi: 10.1371/journal.pmed.1003111 (PMC7228042; doi:10.1371/journal.pmed.1003111)

**S1 Fig.** Study flowchart.

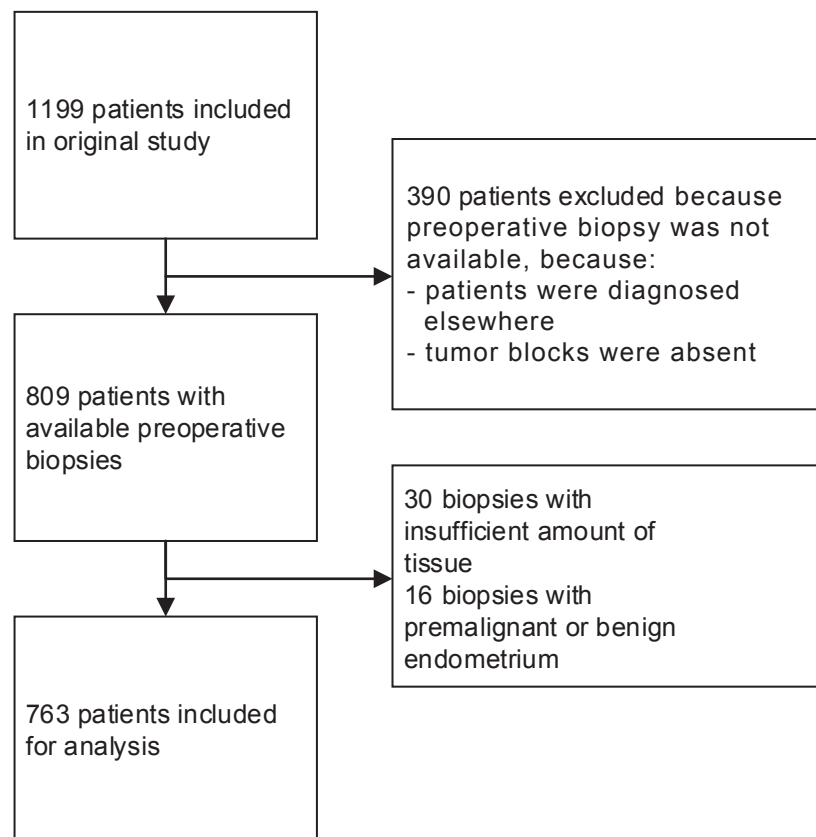

Supplement: S1 Fig — (PDF) [file pmed.1003111.s003.pdf]
