# Supplementary material for: Preoperative risk stratification in endometrial cancer (ENDORISK) by a Bayesian network model: A development and validation study
Source: PLoS Med. 2020 May 15;17(5):e1003111. doi: 10.1371/journal.pmed.1003111 (PMC7228042; doi:10.1371/journal.pmed.1003111)
Supplement: S2 Fig — Decision curves for (A) LNM in the MoMaTEC cohort, (B) 5-year DSS in the MoMaTEC cohort, and (C) 5-year DSS in the PIPENDO cohort. DSS, disease-specific survival; LNM, lymph node metastasis; MoMaTEC, Molecular Markers in Treatment in Endometrial Cancer; PIPENDO; PIpelle Prospective ENDOmetrial carcinoma. (PDF) [file pmed.1003111.s004.pdf]

S2 Figure. Decision curves for (A.). lymph node metastasis in the MoMaTEC cohort, (>). 5-year disease-specific survival in the MoMaTEC cohort, and (C). 5-year disease-specific survival in the PIPENDO cohort.

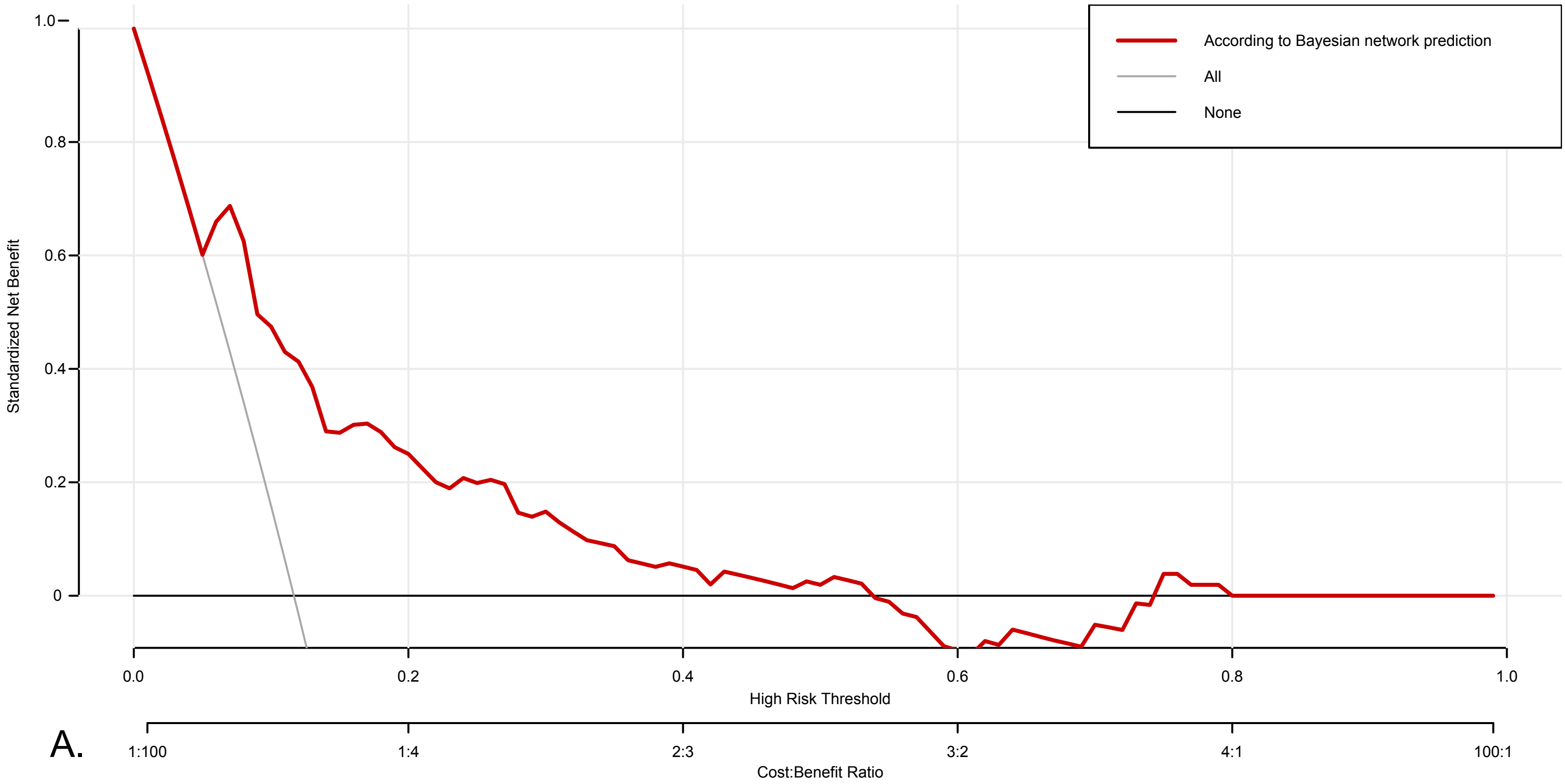

A.

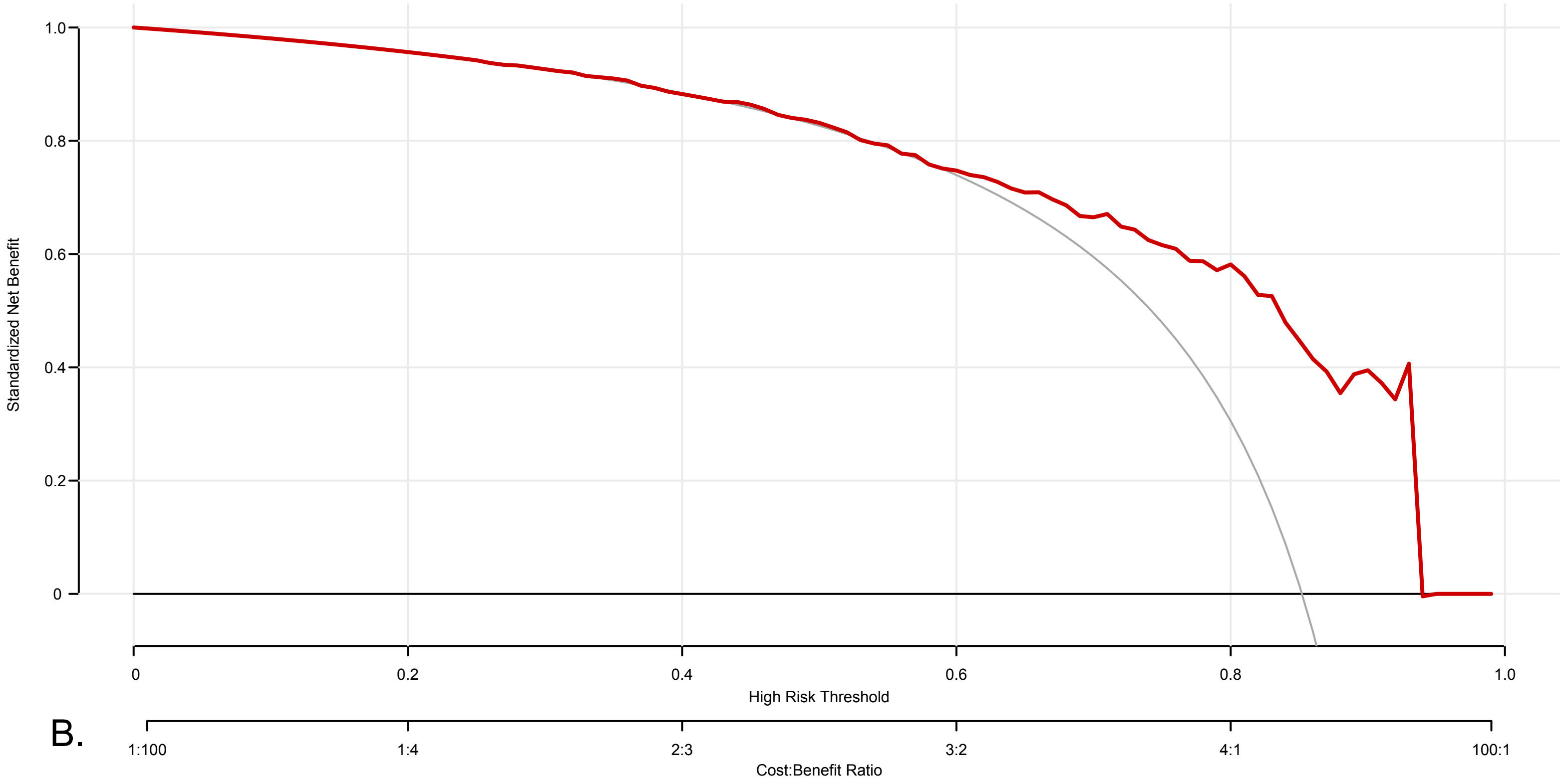

B.

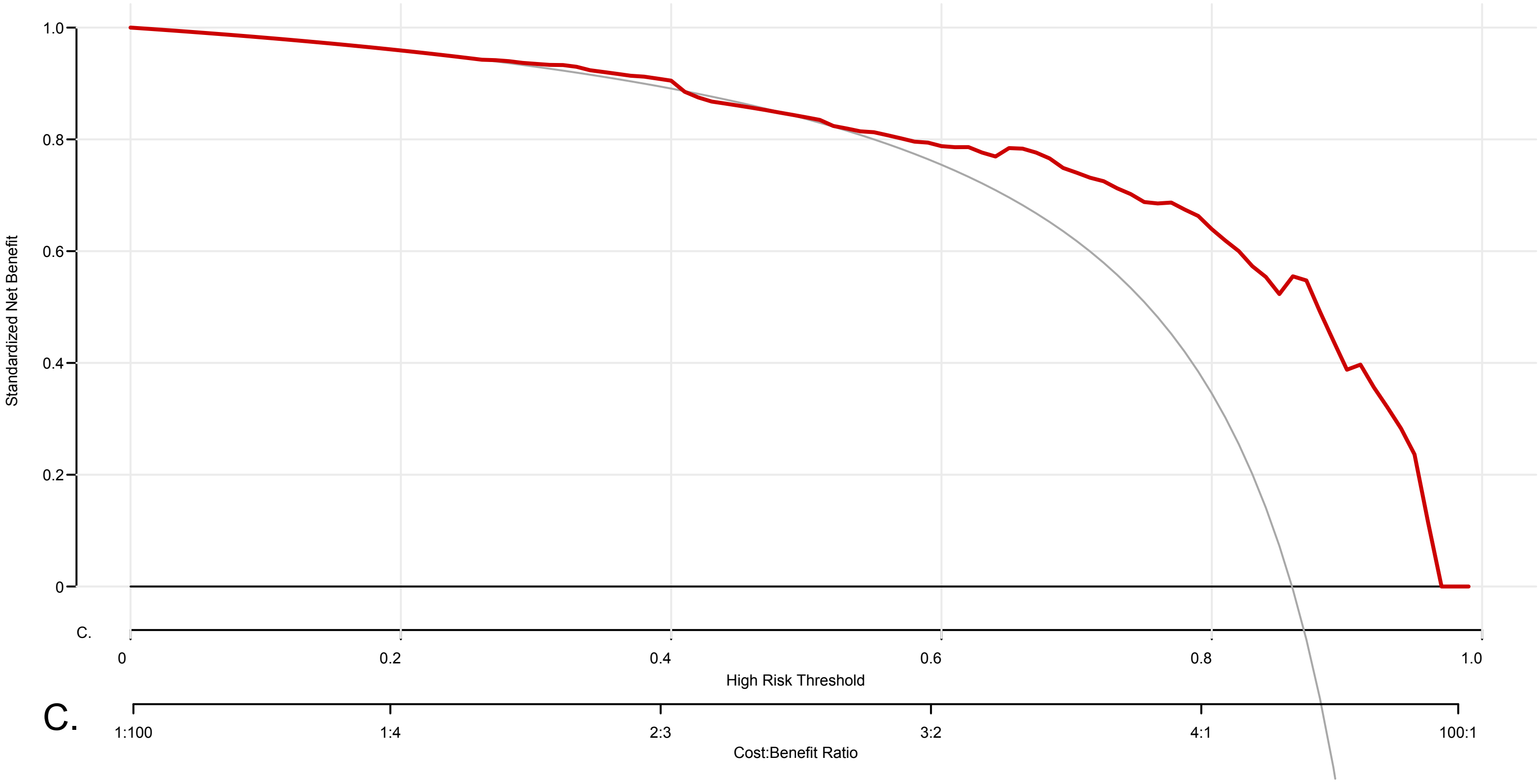

1.0
0.8
0.6
0.4
0.2
0
Standardized Net Benefit
C.
0
0.2
0.4
0.6
0.8
1.0
High Risk Threshold
1:100
1:4
2:3
3:2
4:1
100:1
Cost:Benefit Ratio

C.
