# Supplementary material for: Preoperative risk stratification in endometrial cancer (ENDORISK) by a Bayesian network model: A development and validation study
Source: PLoS Med. 2020 May 15;17(5):e1003111. doi: 10.1371/journal.pmed.1003111 (PMC7228042; doi:10.1371/journal.pmed.1003111)
Supplement: S1 Table — (PDF) [file pmed.1003111.s005.pdf]

S1 Table. Baseline characteristics of included vs. excluded patients.

Variable	Included patients	Excluded patients	P
Age (y)	65 (58-71)	63 (55 – 70)	0.056
Follow-up (months)	60 (45-74)	62 (54 – 86)	0.775
Tumor grade			
1	317 (41.5)	163 (35.4)	0.104
2	289 (37.9)	188 (40.8)	
3	157 (20.6)	110 (23.9)	
Histological subtype			
EEC	714 (93.6)	413 (89.6)	0.090
NEEC	49 (6.4)	48 (10.4)	
Myometrial invasion			
<50%	477 (62.8)	278 (60.8)	0.507
>50%	283 (37.2)	179 (39.2)	
Unknown	3	4	
Cervical invasion			
no	591 (87.3)	337 (80.8)	0.003
yes	86 (12.7)	80 (19.2)	
unknown	86	44	
FIGO stage			
IA	428 (56.1)	256 (55.5)	0.438
IB	196 (25.7)	98 (21.3)	
II	51 (6.7)	37 (8.0)	
IIIA	20 (2.6)	14 (3.0)	
IIIB	4 (0.5)	2 (0.4)	
IIIC	43 (5.6)	39 (8.5)	
IV	19 (2.5)	15 (3.2)	
LVSI			
no	435 (81.9)	274 (79.2)	0.196
yes	96 (18.1)	72 (20.8)	
unknown	232	114	
Lymph nodes			
negative	440 (89.2)	313 (86.5)	0.065
positive	53 (10.8)	49 (13.5)	
unknown	270	50	

Continuous variables are presented as median (with inter-quartile range).
EEC, endometrioid endometrial carcinoma; LVSI, lymphovascular space invasion; NEEC, non-endometrioid endometrial carcinoma.
