# Supplementary material for: Preoperative risk stratification in endometrial cancer (ENDORISK) by a Bayesian network model: A development and validation study
Source: PLoS Med. 2020 May 15;17(5):e1003111. doi: 10.1371/journal.pmed.1003111 (PMC7228042; doi:10.1371/journal.pmed.1003111)
Supplement: S2 Table — LNM, lymph node metastasis. (PDF) [file pmed.1003111.s006.pdf]

S2 Table. Probability estimates for lymph node metastasis given different evidence situations

Evidence provided to the Bayesian network	Lymph node metastasis (%)
No evidence	8.6
Preoperative grade	
1	4.7
2	8.1
3	21.6
Preoperative grade, L1CAM	
1, negative	4.2
1, positive	21.6
2, negative	6.6
2, positive	28.2
3, negative	17.8
3, positive	33.0
Preoperative grade, molecular profile	
1, favorable*	2.6
1, unfavorable†	39.5
2, favorable	4.6
2, unfavorable	39.6
3, favorable	16.1
3, unfavorable	40.0
Preoperative grade, Ca-125	
1, normal	1.5
1, elevated	29.3
2, normal	2.6
2, elevated	40.3
3, normal	7.5
3, elevated	66.8

*Favorable: all IHC stainings were normal (ER, PR positive, L1CAM negative, p53 wildtype).

†Unfavorable (ER, PR negative, L1CAM positive, p53 mutant).

Ca-125, cancer antigen 125; ER, estrogen receptor; L1CAM, L1 cell adhesion molecule; PR, progesterone receptor.
