# Supplementary material for: Preoperative risk stratification in endometrial cancer (ENDORISK) by a Bayesian network model: A development and validation study
Source: PLoS Med. 2020 May 15;17(5):e1003111. doi: 10.1371/journal.pmed.1003111 (PMC7228042; doi:10.1371/journal.pmed.1003111)
Supplement: S3 Table — LNM, lymph node metastasis; MoMaTEC, Molecular Markers in Treatment in Endometrial Cancer. (PDF) [file pmed.1003111.s007.pdf]

S3 Table. Diagnostic accuracy values for the prediction of lymph node metastasis in the MoMaTEC cohort, using various cut-off values

MoMaTEC lymph node metastasis						
Cut-off	Sensitivity	Specificity	PPV	NPV	Predicted positive (%)	FNR
1%	1.00	0.06	0.12	1.00	422 (94.6)	0/24 (0)
5%	0.90	0.62	0.24	0.98	197 (44.2)	4/249 (1.6)
10%	0.75	0.73	0.27	0.96	146 (32.7)	13/300 (4.3)
15%	0.65	0.80	0.30	0.95	113 (25.3)	18/333 (5.4)
20%	0.50	0.84	0.29	0.93	92 (20.6)	25/354 (7.1)
25%	0.48	0.89	0.37	0.93	70 (15.7)	27/376 (7.2)

MoMaTEC, Markers for the Treatment of Endometrial Cancer; PPV, positive predictive value; NPV, negative predictive value; FNR, false negative rate.
