# Supplementary material for: Preoperative risk stratification in endometrial cancer (ENDORISK) by a Bayesian network model: A development and validation study
Source: PLoS Med. 2020 May 15;17(5):e1003111. doi: 10.1371/journal.pmed.1003111 (PMC7228042; doi:10.1371/journal.pmed.1003111)
Supplement: S4 Table — (PDF) [file pmed.1003111.s008.pdf]

S4 Table. Sensitivity analysis for external validation, omitting clinical variables and molecular markers

Clinical and histological markers	MoMaTEC		PIPENDO
	LNM	5-year DSS	5-year DSS
AUC (95%-CI)	0.77 (0.70 – 0.85)	0.76 (0.69 – 0.83)	0.82 (0.76 – 0.88)
Brier score	0.10	0.12	0.10
Predicted *N* of events	51.6	411.4	344.7
Observed *N* of events	52	380	350.5
Predicted/observed ratio (95%-CI)	0.99	1.08	0.98

Molecular and histological markers	MoMaTEC		PIPENDO
	LNM	5-year DSS	5-year DSS
AUC (95%-CI)	0.66 (0.57 – 0.74)	0.76 (0.70 -0.82)	0.79 (0.73 – 0.86)
Brier score	0.10	0.12	0.11
Predicted *N* of events	43.3	410.9	350.5
Observed *N* of events	52	380	330
Predicted/observed ratio (95%-CI)	0.83	1.08	1.06
